# Fungal research in Japan: tradition and future

**DOI:** 10.1186/s40694-020-00104-1

**Published:** 2020-09-21

**Authors:** Norio Takeshita

**Affiliations:** Microbiology Research Center for Sustainability (MiCS), Faculty of Life and Environmental Sciences, University of Tsukuba, Tokyo, Japan

“Washoku” means traditional Japanese cuisine and the dietary culture [1, Tokyo]: it was added to UNESCO Intangible Cultural Heritage list in 2013 and attracts increasing attention globally. Shoyu (soy sauce) and miso (soy-bean paste) are used as fundamental seasonings for Japanese dishes. In addition, the traditional Japanese alcoholic beverage sake is primarily paired with Japanese foods. The filamentous fungus *Aspergillus oryzae* has been used in the production of traditional fermented foods such as shoyu and miso and drinks such as sake for more than 1000 years in Japan, making it the most important filamentous fungus in the country [2] (Fig. 1). *A. oryzae* has even been heralded as the “national microorganism” of Japan [3, 4]. The utility and safety of *A. oryzae* are guaranteed by the long history of use in food fermentation industries and modern molecular analyses [5, 6]. Here, this *Fungal Biology and Biotechnology* special issue collected two reviews and three articles from fungal research in Japan, covering the tradition and future of Japanese fungal biology and biotechnology, using *A. oryzae* as an example.

**Fig. 1:**
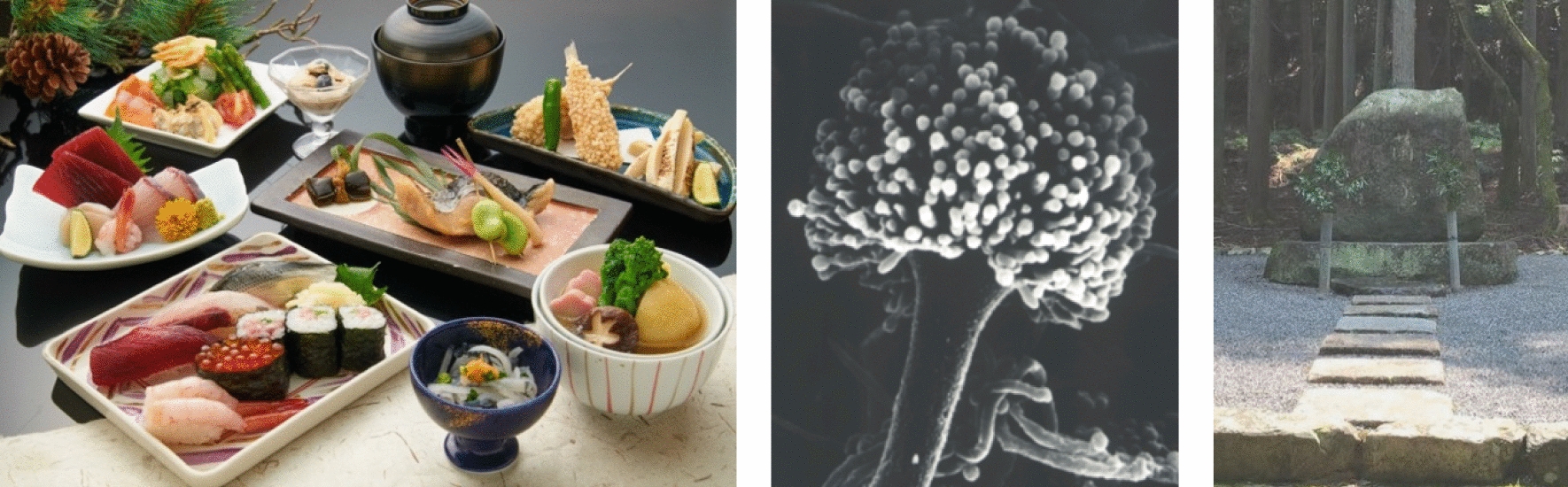
Washoku (left), an electron microscopy image of an *A. oryzae* conidiophore (middle), Microbe Mound (right) at Manshuin Temple in Kyoto; we pay our deepest respect to the innumerable souls of microbes who have dedicated and sacrificed for us

Amylolytic enzymes play essential roles in the fermentation in *A. oryzae.* The production of amylolytic enzymes is induced by starch or malto-oligosaccharides, whereas the expression of amylolytic enzymes is repressed by glucose due to carbon catabolite repression. Among amylolytic enzymes, glucoamylases generate glucose from starch. Prof. Gomi at Tohoku University, one of those whom established molecular biology in *A. oryzae,* has revealed regulatory mechanisms for amylolytic gene expression in *A. oryzae.* His review focuses on fungal research history in Japan and the role of transcription factors in the amylolytic gene expression (in preparation).

One of the distinctive features in the use of *A. oryzae* in the traditional fermentation is the use of solid-state cultivation. “Rice *koji*” is a solid culture of *A. oryzae* on steamed rice grains. Multiple parallel fermentations, wherein saccharification of rice by *A. oryzae* and alcohol fermentation by the budding yeast (*Saccharomyces cerevisiae*) occur simultaneously, lead to the formation of a variety of ingredients in Japanese sake with its characteristic tastes. Sake quality affected by the metabolite composition depends on the combination of raw materials and sake-making parameters (e.g., rice races, rice polishing ratio, water quality, *koji* mold, yeast strains, sake mash fermentation methods) used during manufacturing [7]. In sake brewing, the degree of hyphal penetration into the steamed rice highly correlates with its digestibility, since the hyphae growing into the rice secrete amylases and digest the starch. Yasui and colleagues in Assoc. Prof. Takeshita’s group at the University of Tsukuba visualized mycelial distribution of GFP-tagged *A. oryzae* in rice *koji* made with different types of rice (Fig. 2). In addition, they found that *A. oryzae* increases the nuclear number drastically in the course of 1–3 days cultivation, 20 to more than 200 nuclei in the hyphal tip compartment.

**Fig. 2:**
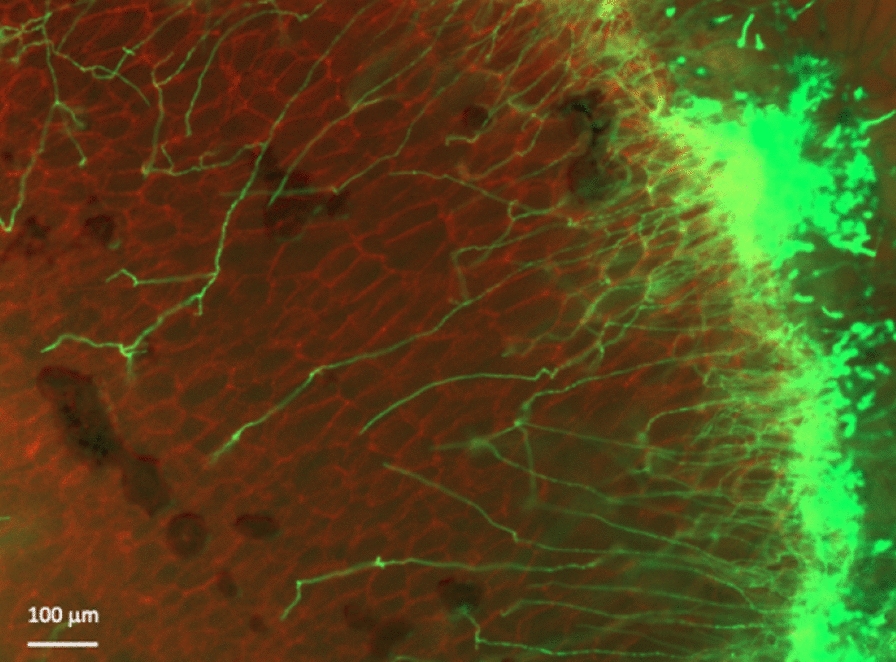
Invasive growth of *A. oryzae* (green) into rice (red)

*A. oryzae* has been used commercially as a host for homologous and heterologous protein production as well in modern biotechnology [8]. Huynh with others in Assoc. Prof. Maruyama’s group at the University of Tokyo, designed *A. oryzae* to produce a monoclonal antibody, which gathers the high demand due to its pivotal role in the current therapeutic application (Fig. 3). The study also gives the possibility of CRISPR/Cas9-based glycoengineering for mimicking the humanized *N*-glycan structure, which is related to another favored function as an immune effector. The article demonstrates *A. oryzae* as an alternative low-cost platform for biopharmaceutical production such as human antibodies, which may expand the industrial application of this filamentous fungus beyond Japan by offering additional treatment options to patients over the world.

**Fig. 3:**
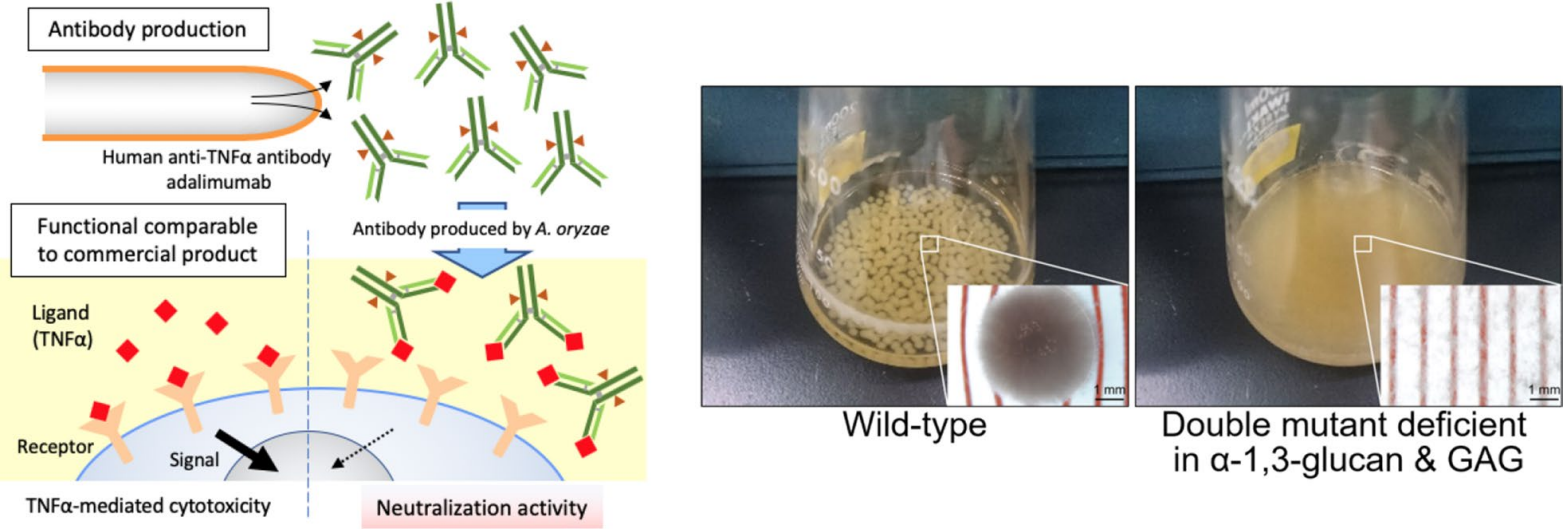
Modifications to *A. oryzae* for biotechnology purposes. Engineering *A. oryzae* to produce a monoclonal antibody (left). Comparison between *A. oryzae* hyphal pellets produce by wild type vs the dispersed hyphae produced by a mutant (right)

The importance of cell wall α-1,3-glucan, a major cell wall polysaccharide, as a virulence factor was reported in human and plant pathogenic species, whereas universal function of α-1,3-glucans has also been demonstrated as an aggregation factor [9]. Filamentous fungi generally form aggregated hyphal pellets in liquid culture, which reduces cell density and fermentation productivity, however the α-1,3-glucan-deficient mutants of *Aspergillus nidulans* did not form hyphal pellets but fully dispersed. Besides α-1,3-glucan, an extracellular matrix polysaccharide galactosaminogalactan (GAG) is also involved in the aggregation in *A. oryzae* [10] (Fig. 3, right). Miyazawa and colleagues in Prof. Abe’s group at Tohoku University, overview its biological functions and biosynthesis, and discuss the industrial applications of fungi deficient in aggregation for high-cell-density cultivation, which is effective for high productivity in industrial fermentation.

Developing antibiotics and antifungal agents is an important strategy to fight against infectious diseases, and fungal peptidyl compounds are a rich resource of such compounds; e.g. penicillin, cyclosporin and micafungin. Fungal peptidyl compounds are mainly classified into two types; non-ribosomal peptides (NRPs) and ribosomally synthesized and post-translationally modified peptides (RiPPs). RiPPs are a relatively new class of secondary metabolites produced by microorganisms; the RiPP backbone structure is cleaved out from a corresponding precursor peptide for further modification such as methylation and cyclization. Umemura and colleagues at AIST (National Institute of Advanced Industrial Science and Technology), have found a new type of fungal RiPP biosynthetic pathway [11, 12]. The genes encoding precursor peptides (FRiPS factors) are widely conserved in the kingdom Fungi. The diversity of FRiPS factors and the relation with mating factors and other sexual hormones are analyzed by bioinformatics analyses (Fig. 4).

**Fig. 4:**
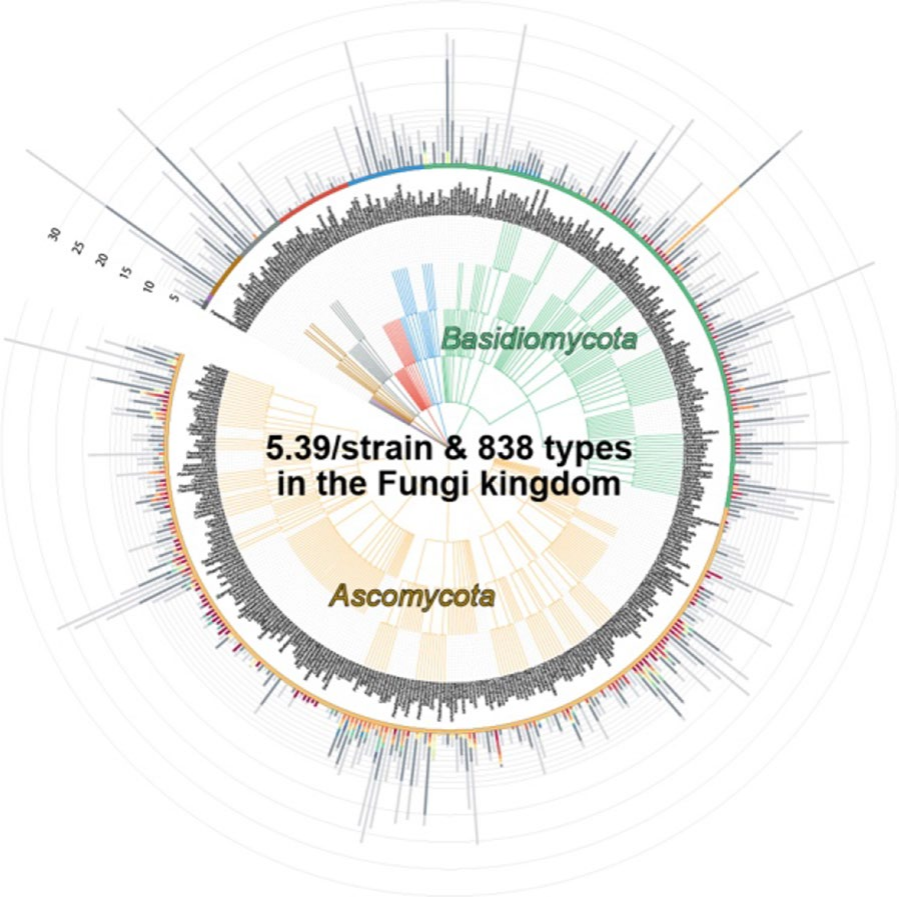
Diversity of FRiPS factors in the kingdom Fungi

*A. oryzae* is widely used for secondary metabolite production by heterologous expression. The same group, Umemura et al. recently reported promoter tools for further development of *A. oryzae* as a platform for fungal secondary metabolite production [13]. They surveyed for constitutively highly expressed genes among the 83 transcriptome datasets and found promoter sequences that can be used as heterologous expression tools in *A. oryzae.*

Humankind has been producing fermented foods long before correctly recognizing the existence of the microorganisms by utilizing the microorganisms that match the land topography and climate. The presence of microorganisms may affect not only local eating habits but also lifestyle religious views, etc. *A. oryzae* has been bred in the history of brewing for over 1000 years and plays a fundamental role in Japanese society and industry. Fermentation technologies, which have been refined over the years, form the basis of microbial biotechnology. These five articles highlight that there is still much to be learned or exploited about this fungus.

## References

[1] https://www.maff.go.jp/e/japan_food/washoku/pdf/wasyoku_english.pdf.

[2] Machida M, Yamada O, Gomi K (2008). Genomics of *Aspergillus oryzae*: learning from the history of *Koji* mold and exploration of its future. DNA Res.

[3] Kitamoto K (2015). Cell biology of the koji mold *Aspergillus oryzae*. Biosci Biotechnol Biochem.

[4] Ichishima E (2016). Development of enzyme technology for *Aspergillus oryzae*, *A. sojae*, and *A. luchuensi*s, the national microorganisms of Japan. Biosci Biotechnol Biochem..

[5] Machida M (2005). Genome sequencing and analysis of *Aspergillus oryzae*. Nature.

[6] Kiyota T, Hamada R, Sakamoto K, Iwashita K, Yamada O, Mikami S (2011). Aflatoxin non-productivity of *Aspergillus oryzae* caused by loss of function in the *aflJ* gene product. J Biosci Bioeng.

[7] Yazawa H, Tokuoka M, Kozato H, Mori Y, Umeo M, Toyoura R, Oda K, Fukuda H, Iwashita K (2019). Investigation of relationship between sake-making parameters and sake metabolites using a newly developed sake metabolome analysis method. J Biosci Bioeng.

[8] Christensen T, Woeldike H, Boel E, Mortensen SB, Hjortshoej K, Thim L, Hansen MT (1988). High level expression of recombinant genes in *Aspergillus oryzae*. Nat Biotech..

[9] Yoshimi A, Miyazawa K, Abe K (2017). Function and biosynthesis of cell wall α-1,3-glucan in fungi. J Fungi (Basel)..

[10] Miyazawa K, Yoshimi A, Sano M, Tabata F, Sugahara A, Kasahara S, Koizumi A, Yano S, Nakajima T, Abe K (2019). Both galactosaminogalactan and α-1,3-glucan contribute to aggregation of *Aspergillus oryzae* hyphae in liquid culture. Front Microbiol..

[11] Nagano N, Umemura M, Izumikawa M, Kawano J, Ishii T, Kikuchi M, Tomii K, Kumagai T, Yoshimi A, Machida M, Abe K, Shin-Ya K, Asai K (2016). Class of cyclic ribosomal peptide synthetic genes in filamentous fungi. Fungal Genet Biol.

[12] Ye Y, Minami A, Igarashi Y, Izumikawa M, Umemura M, Nagano N, Machida M, Kawahara T, Shin-Ya K, Gomi K, Oikawa H (2016). Unveiling the biosynthetic pathway of the ribosomally synthesized and post-translationally modified peptide ustiloxin B in filamentous fungi. Angew Chemie Int Ed..

[13] Umemura M, Kuriiwa K, Dao LV, Okuda T, Terai G (2020). Promoter tools for further development of *Aspergillus oryzae* as a platform for fungal secondary metabolite production. Fungal Biol Biotechnol..

